# Circadian Fluctuation Changes in Intraocular Pressure Measured Using a Contact Lens Sensor in Patients with Glaucoma after the Adjunctive Administration of Ripasudil: A Prospective Study

**DOI:** 10.3390/jpm13050800

**Published:** 2023-05-06

**Authors:** Shih-Kung Huang, Mai Ishii, Yuki Mizuki, Tatukata Kawagoe, Masaki Takeuchi, Eiichi Nomura, Nobuhisa Mizuki

**Affiliations:** 1 Department of Ophthalmology and Visual Science, Yokohama City University Graduate School of Medicine, Yokohama 236-0004, Kanagawa, Japan; 2 Department of Ophthalmology, Yokohama Hodogaya Central Hospital, Yokohama 240-8585, Kanagawa, Japan

**Keywords:** circadian IOP fluctuation, contact lens sensor, glaucoma, intraocular pressure, ripasudil

## Abstract

Nocturnal and circadian intraocular pressure (IOP) fluctuations are important issues in glaucoma treatment. Ripasudil 0.4% eye drops, a new glaucoma medication, lowers IOP by increasing aqueous humor outflow through the trabecular meshwork. We aimed to compare differences between circadian IOP fluctuations measured using a contact lens sensor (CLS) before and after administering 0.4% ripasudil eye drops adjunctively to patients with primary open-angle glaucoma (POAG) and normal tension glaucoma (NTG). Patients with POAG (*n* = 1) and NTG (*n* = 5) underwent 24 h IOP monitoring with a CLS before and after administering ripasudil eye drops every 12 h (8 a.m., 8 p.m.) for 2 weeks without discontinuing currently used glaucoma medications. No vision-threatening adverse event occurred. The reduction in IOP fluctuation and the reduction in the SD of IOP in 24 h, awake time and sleep time did not reach statistical significance. The baseline office-hour IOP, which was measured using Goldmann applanation tonometry (GAT), ranged in the low teens, and the reduction in office-hour IOP also did not show a significant difference. Further study is necessary to evaluate whether the low baseline IOP with less IOP reduction relates to attenuated IOP fluctuation reduction.

## 1. Introduction

Glaucoma refers to a group of ocular diseases that can result in optic nerve fiber layer loss leading to constriction of the visual field. Glaucoma is a major cause of irreversible blindness globally. Lowering the intraocular pressure (IOP) remains the most important treatment for glaucoma [1]. Despite achieving good control of IOP, deterioration of the visual field may still be observed in routine clinical practice. Determining whether IOP other than that measured during office hours is well controlled remains a challenge [2]. Measuring IOP several times a day using Goldmann applanation tonometry (GAT) or non-contact pneumotonometry may solve this problem [3,4]. However, GAT and non-contact pneumotonometry are measured in the sitting position, and body position may affect changes in IOP. Moreover, the limited frequency of measurements poses a challenge to the detection of any change in IOP [5]. A contact lens sensor (CLS) can continuously measure the IOP for the 24 h in a day and in the supine position while sleeping; therefore, it could solve this problem [6].

Ripasudil (Glanatec^®^ ophthalmic solution, 0.4%; Kowa Company, Ltd., Nagoya, Japan) is a Rho-associated coiled-coil-containing protein kinase inhibitor drug, and in Japan, it was approved for glaucoma treatment in 2014. Ripasudil lowers IOP by inducing a morphological change in the trabecular meshwork (TM) and then increasing the conventional aqueous outflow through the TM and Schlemm’s canal [7]. The IOP-lowering effect of ripasudil has been demonstrated in previous studies [8]. Tanihara et al. reported that the IOP-lowering effect of ripasudil persisted for 7 h after the first (9 a.m.) and second (9 p.m.) administrations [9]. Moreover, ripasudil was more effective in lowering IOP during the day and at night than the placebo. However, the IOP was measured in a sitting position using GAT, and sleep was disrupted during the measurement. The nocturnal IOP-lowering effect of ripasudil in the supine position can be measured using a CLS without disrupting sleep; therefore, we can acquire information regarding the IOP fluctuation change after ripasudil eye drop administration under natural physiological conditions.

To the best of our knowledge, there has been no research to date, and this is the first study to compare the 24 h IOP-fluctuation changes before and after a 2-week adjunctive administration of ripasudil eye drops using a CLS in patients with glaucoma.

## 2. Materials and Methods

### 2.1. Patients

This was a prospective open-label, single-arm study. Between January 2022 and April 2023, one patient with primary open-angle glaucoma (POAG) and five patients with normal tension glaucoma (NTG) from the Yokohama Hodogaya Central Hospital, Japan, a co-sponsor institute of this study, were enrolled in this study. The inclusion criteria required the patients to be aged 20 years or older and diagnosed with POAG or NTG. The diagnostic requirements for a glaucomatous visual field included the following: (1) a glaucoma hemifield test result outside the normal limits, compatible with retinal nerve fiber layer (RNFL) loss; (2) three or more abnormal points with a probability of being normal of *p* < 5% and at least one point with a pattern deviation of *p* < 1%; or (3) a pattern standard deviation (SD) of *p* < 5%. Patients were excluded if (1) their condition was diagnosed as secondary open-angle glaucoma or angle-closure glaucoma, (2) they had any history of silicone allergy or adverse reactions to contact lens use before, or (3) they received systemic steroid treatment or topical steroid eye drops treatment for systemic disease or uveitis. The patients underwent a complete ophthalmic examination during the screening visit, including slit-lamp biomicroscopy, gonioscopy, GAT, refraction tests, central corneal thickness (CCT) measurement, dilated fundus examination, standard automated perimetry (Humphrey 30-2 SITA-Standard; Carl Zeiss Meditec, Inc., Dublin, CA, USA), and RNFL thickness examination (SS-OCT; DRI OCT Triton-1, Topcon, Tokyo, Japan). The eye with the worst mean deviation in the visual field test was recruited. The baseline office-hour IOP was measured using GAT, and the 24 h IOP was measured using a CLS without hospital admission. The CLS was administered by an ophthalmologist in the ophthalmology outpatient department. The patients were instructed to record the time of sleep and the time of awakening. The office-hour GAT IOP was measured before applying a CLS, and then CLS 24 h IOP measurements were repeated at around the same time in the day as the first CLS measurement after the 2-week administration of ripasudil eye drops every 12 h (8 a.m., 8 p.m.) without discontinuing the currently used eye drops.

### 2.2. Parameters of Contact Lens Sensor Measurements

**Figure 1:**
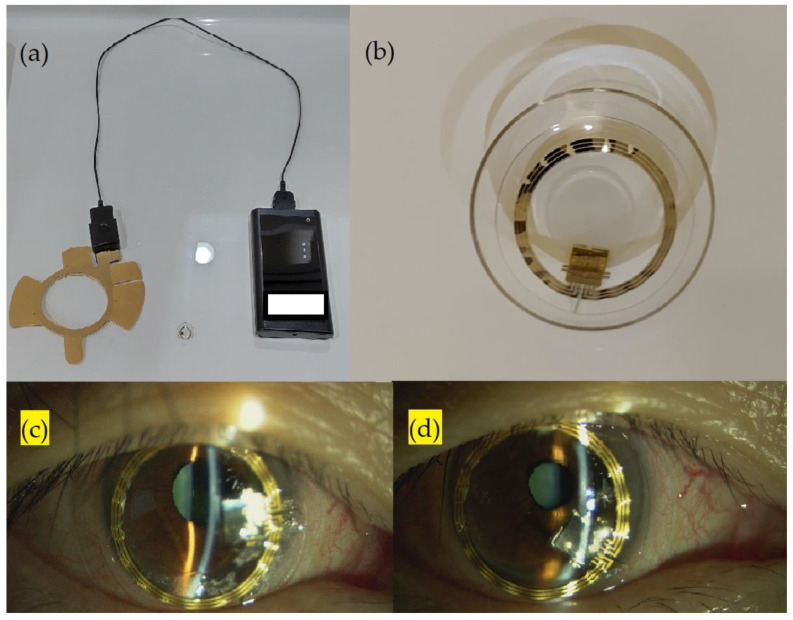
External photograph of CLS (**a**) antenna and recorder; (**b**) contact lens; (**c**) external eye photograph of the participant’s ocular surface right after CLS administration; (**d**) external eye photograph after 24 h administration, mild conjunctiva congestion noted.

The SENSIMED Triggerfish^®^ (Sensimed AG, Lausanne, Switzerland) comprises a wireless silicon CLS with embedded strain gauges, an antenna to be attached around the eye, and a recorder. A photograph of the CLS is presented in Figure 1. The CLS device can record IOP continuously for 24 h without disturbing the patient’s daily routine [10]. The CLS records the IOP-related corneoscleral biodimensional change. However, the output is provided in millivolts equivalent (mVeq), which cannot be converted to IOP in millimeters of mercury (mmHg) directly. A highly positive correlation has been observed between the IOP measured using a CLS (mVeq) and that measured using a tonometer (mmHg) [11,12]. Otherwise, another study reported a low correlation between the IOP measured using a CLS (mVeq) and the IOP (mmHg) measured using the Tono-pen^®^ XL applanation tonometer (r = 0.291) [13]. The value of IOP measured using a CLS (mVeq) could not be considered as the true IOP, but the fluctuation of the IOP value measured using a CLS is considered effective and safe for clinical use and has been used in some clinical projects pertaining to glaucoma research [14].

**Figure 2:**
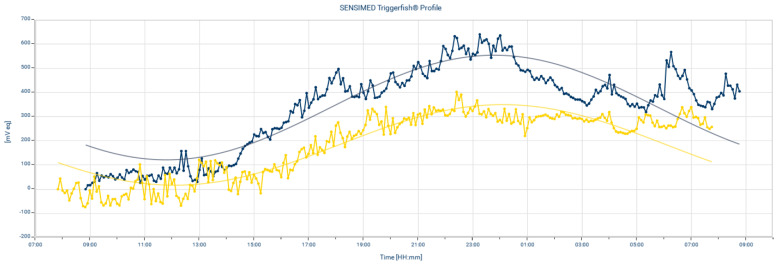
Example of the 24 h intraocular pressure-related pattern before and after ripasudil eye drop administration. Contact lens sensor (CLS) pattern before (blue tracing) and after (yellow tracing) administration. Smooth curve: cosine fit curve. Polygonal line: CLS raw data.

The CLS parameters included the average and SD values of IOP measurements in a 24 h day, at sleep and awake times; IOP fluctuations (difference between maximum value and minimum value) during the 24 h, at sleep and awake times; maximum value during the 24 h, at sleep and awake times; difference between the sleep and awake average IOPs; amplitude of the cosine fit curve; and IOP in mmHg measured using GAT. The CLS measured the IOP every 5 min; therefore, data (ID) were recorded 288 times within 24 h. The SD values, IOP fluctuations, maximum values for 24 h and sleep and awake times were calculated using RAW data. The circadian rhythm measurement was based on the cosine fit curve used to study the circadian biological rhythm. The cosine fit curve of the 24 h IOP measurements was examined using JMP statistical software with time-series analysis. The AUC was calculated using the integral of the cosine fit curve during sleep. To calculate the mean IOP of sleep time, the sleep time AUC was divided by the ID interval number of sleep time. To compare the circadian IOP fluctuations before and after the administration of ripasudil eye drops, the amplitudes of the cosine fit curve and the difference between the sleep time and awake time average IOPs were calculated. Ripasudil eye drops were administered at 8 a.m. and 8 p.m. According to Tanihara et al., the IOP-lowering effect of ripasudil persists for approximately 7 h after administration [9]. Therefore, in order to avoid the period that ripasudil might no longer be effective, we divided the awake time and sleep time IOP measurement period into two halves starting at 3 p.m. and 3 a.m., respectively, and SD values and IOP fluctuations of the first half in awake time and sleep time were calculated. A sample CLS measurement is presented in Figure 2.

### 2.3. Statistical Analysis

The paired samples were compared using paired *t*-tests. Except where stated otherwise, the data are presented as mean ± SD values. All analyses were performed using JMP^®^ PRO 15 (SAS institute, Inc., Cary, NC, USA 2019).

### 2.4. Ethical Approval

The ethics committee of the Yokohama City University Graduate School of Medicine approved the study, and written informed consent was obtained from all participants. The study adhered to the contents of the Declaration of Helsinki. The study was registered (UMIN000041093) in the University Hospital Medical Information Network Clinical Trials Registry (UMIN-CTR) of Japan.

## 3. Results

The study included one patient with POAG and five patients with NTG. The ocular characteristics and demographic features of the participants are summarized in Table 1. None of the patients withdrew their participation in the study due to the adverse effects of ripasudil. Transient conjunctival congestion and blurred vision were noted in all cases after the CLS examination; however, these symptoms improved within one day. No other adverse effects, such as corneal ulcers, were noted.

The results of the paired *t*-tests before and after the administration of ripasudil are presented in Table 2. Although the value of each parameter became lower after ripasudil eye drop administration, the reduction did not reach statistical significance. The IOP measured in mmHg using GAT also did not reveal statistical significance.

## 4. Discussion

The lowering of nocturnal IOP plays a crucial role in the daily treatment of glaucoma, and the nocturnal IOP-lowering effects of glaucoma medications have been reported in previous studies. β-blockers (e.g., timolol) are less effective in reducing IOP during nighttime hours [15,16,17]. The efficacy of carbonic anhydrase inhibitors (CAIs) in lowering nocturnal IOP remains controversial, as a significant reduction in nocturnal IOP was reported by some studies [18,19], whereas ineffectiveness in lowering IOP during the nocturnal hours was reported by another study [20]. β-blockers and CAIs lower IOP by suppressing aqueous humor production. A previous study revealed the presence of a circadian rhythm in aqueous humor production; the aqueous humor production is lower at night, and this might make the IOP-lowering effect of β-blockers and CAIs less effective at night [21]. Prostaglandin (PG) analogs lower IOP by affecting the uveoscleral outflow. Notably, among PG analogs, different medications possess varying nocturnal IOP-lowering effects, even with similar IOP-lowering mechanisms. Stewart et al. reported that for latanoprost, the mean reduction in nighttime IOP was significantly lower than that of daytime IOP. However, the same effect could not be noted for bimatoprost and travoprost [22]. Ripasudil reduces the IOP by changing the cell morphology of the TM. The IOP-lowering effect would presumably be less affected by the circadian rhythm of aqueous humor production. Therefore, theoretically, there might be a chance to reduce the nocturnal IOP and then reduce the IOP circadian fluctuation after ripasudil eye drop administration. However, the reduction in circadian fluctuations did not reach statistical significance after a 2-week ripasudil eye drop administration in the present study. The possible reason is that the nocturnal IOP reduction amount was not significantly larger than the diurnal IOP reduction after ripasudil eye drop administration. However, there is no study comparing the reduction in nighttime IOP with that of daytime IOP after ripasudil eye drop administration directly. Further evaluation is necessary.

Due to the effect of the loosening of the TM and the juxtacanalicular tissue, the mechanism of the IOP-lowering effect of ripasudil is considered similar to that of selective laser trabeculoplasty (SLT). Previous studies reported the relationship, comparisons, and treatment effects of ripasudil and SLT. Baba et al. reported increased success rates of SLT in patients with ripasudil-effective glaucoma [23]. Ono et al. reported that both adjuvant ripasudil and SLT reduced IOP significantly in patients with inadequately controlled glaucoma, and the percentage reduction in IOP between the two groups was not statistically significant [24]. Tojo et al. used a CLS to measure the changes in IOP before and after SLT in patients with NTG [25]. They reported that the reduction in circadian IOP fluctuation before and after SLT was not statistically significant (*p* = 0.77); however, the reduction in the nocturnal IOP fluctuation was significant (before SLT, 290 ± 86 mVeq vs. after SLT, 199 ± 31 mVeq; *p* = 0.014). In the present report, the reduction in circadian IOP fluctuation, sleep time IOP fluctuation, IOP fluctuation of the first half of sleep time and reduction in IOP measured by GAT were all not statistically significant. The baseline IOP and the characteristics of patients were not similar between the previous reports and the present study, so we could hardly compare the IOP-fluctuation-reducing effect between SLT and ripasudil directly. Whether there is similarity in IOP lowering-mechanisms in regard to IOP fluctuation changes needs further evaluation.

The amplitude of the cosine fit curve is the parameter which is considered the circadian IOP fluctuation, and the reduction in amplitude is considered as an important factor for glaucoma control. However, some controversial results have been reported. Hoban et al. reported a positive trend between higher amplitude and visual field progression without statistical significance (*p* = 0.053) [26]. Tojo et al. reported that the amplitude of the cosine fit curve did not show a positive correlation with a rapid progression visual field, but a positive correlation did exist with the nocturnal maximum value, nocturnal IOP fluctuation (the difference of nocturnal maximum and minimum value) and SD value. Moreover, the SD value of IOP was the CLS parameter which was most associated with the rapid progression of visual field changes (24 h IOP SD, *p* = 0.0404; diurnal IOP SD, *p* = 0.0330; nocturnal IOP SD, *p* = 0.0027) [27]. SD represents the amount of dispersion in a dataset. Therefore, the CLS SD indicates the IOP fluctuation and is different from circadian fluctuation. The lower the value of SD, the more stable the IOP is. The findings of this previous study could imply that a constant and stable IOP at any time in a day is important for glaucoma treatment. The SD of the 24 h IOP measured by the CLS in patients with NTG was greater than that of healthy individuals (patients with NTG, 112.51 ± 26.90 mVeq vs. healthy individuals, 85.18 ± 29.61 mVeq; *p* = 0.002). Moreover, the sleep-time IOP SD was significantly lower than the awake-time IOP SD in patients with NTG [28]. The baseline SD values of the present study were quite similar with those of a previous report. Moreover, a lower IOP with larger IOP SD during awake time and higher IOP with lower IOP SD during sleep time were observed both in the present and previous studies [27,28]. According to the Goldmann equation, episcleral venous pressure (EVP) variation may affect the IOP variation. Heavier daily physical movements and a wider range of blood pressure variation during awake time could explain the large awake-time IOP SD. However, other factors, especially those that could affect sleep-time EVP, remain unclear. The quality of sleep and sleeping behavior may be possible factors; therefore, further studies on how the sleep physiology and autonomic nerve system affect EVP may possibly clarify these aspects. In the present study, the SD values of all the IOP categories were reduced within a day following ripasudil administration; however, no statistically significant difference was noted. To the best of our knowledge, no report focusing on the change in the IOP SD value following any glaucoma treatment exists. For lowering the IOP SD, lowering the IOP might be important; moreover, we believe that different methods other than lowering IOP with updated glaucoma treatments exist for lowering the IOP SD value.

The 24 h average IOP mVeq (*p* = 0.0265) and awake-time average IOP mVeq (*p* = 0.0093) were significantly lower after ripasudil eye drop administration than those before the ripasudil eye drop administration. Although it was not significant, a trend of reduction in sleep-time average IOP mVeq could be noted (*p* = 0.0551). As mentioned above, mVeq is the measurement of biodimensional change between the corneoscleral surface. Whether mVeq could be presented as a real IOP measurement remains controversial. There are some possible reasons that result in the change of corneoscleral surface after ripasudil eye drop administration, such as IOP reduction (IOP lowering effect of ripasudil), conjunctiva hyperemia and increasing vessel density in the sclera (ocular surface events resulted from ripasudil eye drop administration), etc. However, we could not definitively determine the reason. Further evaluation, such as 24 h IOP mmHg measured by tonometry and anterior segment OCT examination, may help in solving this question.

**Figure 3:**
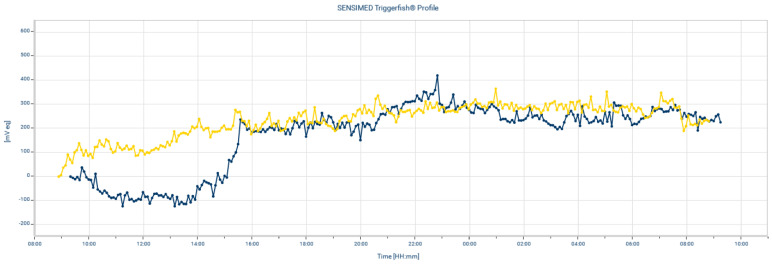
Example of the reproducibility of 24 h intraocular pressure after ripasudil eye drop administration using a CLS. r = 0.841. Blue tracing: first time measurement. Yellow tracing: second time measurement for reproducibility exam.

The reproducibility of CLS remains an important issue. The previous study reported that the overall mean correlation r = 0.59 was noted between the two measurements in the same patients [29]. We repeated the CLS measurements after ripasudil eye drop administration in two patients. A sample of CLS measurement reproducibility is presented in Figure 3. High positive correlations were noted (mean Pearson’s correlation r = 0.854). The difference in the value of the two measurements was calculated and presented in Table 3. The difference value percentage of the awake-time maximum, awake-time fluctuation, sleep-time maximum value, and sleep-time fluctuation between the two measurements were quite similar to the previous report (29.5%, 40.4%, 12.9% and 22.9%, respectively) [27]; therefore, we believed that the validity of the present study was similar to the previous report.

**Figure 4:**
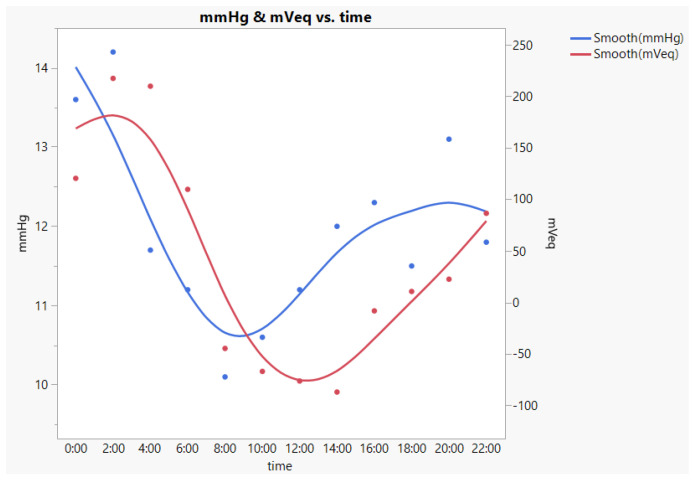
Example of the comparison of IOP (mmHg) using ic200 and IOP (mVeq) using CLS after ripasudil eye drop administration in a participant. r = 0.558. Blue smooth tracing: IOP (mmHg) using ic200. Red smooth tracing: IOP (mVeq) using CLS.

The correlation between CLS IOP and tonometry IOP remains controversial [11,12,13]. In order to measure the IOP (mmHg) in the supine position in the sleep time, we used an i care ic200 to measure the 24 h IOP fluctuation every 2 h in two patients. A sample of CLS IOP (mVeq) and ic200 IOP (mmHg) measurements in a participant after ripasudil eye drops administration is presented in Figure 4. The mean Pearson’s correlation between CLS IOP and ic200 IOP was r = 0.464. However, we could not compare the correlation value with the results of the previous studies directly, due to the different tonometry methods used. The ratio of the circadian fluctuation of IOP measured by the ic200 and IOP measured by the CLS were calculated and presented in Table 4. The ratio between the two cases were similar.

In clinical performance, ripasudil eye drops are often used as a second-line treatment. Therefore, this study was intentionally conducted without discontinuing the currently used eye drops to replicate real-world clinical performance. Tanihara et al. reported increased IOP reduction with a high IOP at baseline [30]. Inazaki et al. reported an IOP reduction of −2.6 mmHg from baseline (baseline IOP: 17.9 ± 4.5 mmHg) after 12 months following treatment in patients with glaucoma receiving the maximum number of medications after adjunctive ripasudil eye drops administration [31]. In the present study, the baseline IOP was lower than those of the previous studies; therefore, a dampened IOP reduction could be expected. Moreover, the reduction in IOP measured by GAT did not reach statistical significance, nor did the IOP fluctuation measured by CLS. Further study is necessary to evaluate whether the attenuated IOP reduction may relate to attenuated IOP fluctuation reduction.

The present study had some limitations. First, owing to the high percentage of transient blurred vision and irritable sensations experienced while wearing the CLS and the high occurrence of conjunctival hyperemia following ripasudil eye drop administration, recruiting patients, especially older adult patients who had no experience of wearing contact lenses, was challenging. Moreover, since our study required the participants to receive CLS measurements twice, recruitment was even more challenging. Therefore, the number of participants was small. Second, a previous study reported that many physiological events, such as body position, blood pressure fluctuations, etc., could affect the relationship between IOP and ocular dimensional measurements [32]. We did not instruct the participants to sleep in a specific position or record the body position during sleeping; however, we had instructed the participants to sleep as they usually sleep in ordinary life to acquire the real IOP in their daily life. Third, the potentially unknown artifacts of CLSs, such as the upward drift phenomenon and contact lens fitting condition, were not considered. The upward drift phenomenon, which occurs after wearing the CLS overnight, results from a change in the corneal curvature [33,34]. How this phenomenon affects the accuracy of CLS measurements remains unclear. However, we conducted the study twice, and compared the data of the patients before and after ripasudil eye drop administration. Therefore, the factors that could possibly affect the validity of CLS, such as the contact lens–cornea fitting conditions, cornea edema, etc., should be consistent in these two measurements and be balanced out. Lastly, contact lenses may act as barriers that affect the absorption of eye drops and weaken the IOP-lowering effect. Therefore, the true effect of eye drops could be larger than that measured using the CLS in this study.

## 5. Conclusions

The reduction in circadian IOP fluctuation, nocturnal IOP fluctuation and SD value of IOP did not reach statistical significance as measured using a CLS after a 2-week adjunctive administration of ripasudil eye drops in patients with glaucoma. The baseline IOP was lower in the present study. The reduction in IOP measured by GAT also did not reach statistical significance. Further study is necessary to evaluate whether the low baseline IOP with less IOP reduction is the reason for the attenuated IOP fluctuation reduction.

## Figures and Tables

**Table 1:** Baseline demographic and ocular characteristics of the study participants.

Characteristic	Value
Age, years (mean ± SD)	58.33 ± 13.89
Sex (n)	
Male	4
Female	2
Ancestry (n)	
Asian	6
SER, D (mean ± SD)	−2.96 ± 1.6
CCT, µm (mean ± SD)	516.5 ± 28.85
IOP, mmHg (mean ± SD)	13.167 ± 2.714
Visual field parameters (mean ± SD)	
MD, dB	−4.78 ± 3.3
PSD, dB	6.56 ± 4.54
VFI, %	88.33 ± 8.31
OCT parameters	
RNFL thickness, µm (mean ± SD)	71.08 ± 9.36
Medication number (mean ± SD)	2 ± 1.09

Abbreviations: CCT, central corneal thickness; D, diopters; IOP, intraocular pressure; MD, mean deviation; OCT, optical coherence tomography; PSD, pattern standard deviation; RNFL, retinal nerve fiber layer; SD, standard deviation; SER, spherical equivalent refraction; VFI, visual field index.

**Table 2:** Comparison of the differences between intraocular pressure-related parameters before and after administering ripasudil eye drops.

CLS Parameter, GAT IOP	Before Ripasudil	After Ripasudil	*p*-Value
24 h average IOP, mVeq	218.760 ± 80.100	102.311 ± 97.784	0.0265 *
24 h IOP fluctuation, mVeq	433.467 ± 135.872	407.867 ± 90.102	0.3337
24 h IOP maximum, mVeq	407.300 ± 147.486	337.150 ± 91.250	0.1331
Sleep time average IOP, mVeq	296.774 ± 106.009	216.554 ± 110.489	0.0551
Sleep time average IOP before 3 a.m., mVeq	297.879 ± 125.821	221.780 ± 125.879	0.0855
Sleep time IOP fluctuation, mVeq	173.817 ± 70.431	146.133 ± 35.187	0.2529
Sleep time IOP fluctuation before 3 a.m., mVeq	166.250 ± 73.625	118.500 ± 28.613	0.1358
Sleep time IOP maximum value, mVeq	396.719 ± 148.986	291.650 ± 114.563	0.0723
Awake time average IOP, mVeq	178.478 ± 67.601	89.808 ± 71.734	0.0093 *
Awake time average IOP before 3 p.m., mVeq	94.717 ± 55.398	31.599 ± 121.857	0.1039
Awake time IOP fluctuation, mVeq	386.400 ± 153.716	370.67 ± 112.953	0.3828
Awake time IOP fluctuation before 3 p.m., mVeq	158.867 ± 44.401	142.033 ± 39.801	0.0647
Awake time IOP maximum value, mVeq	360.217 ± 160.586	299.300 ± 98.133	0.1439
24 h IOP SD, mVeq	105.442 ± 49.723	102.602 ± 36.562	0.4453
Sleep time IOP SD, mVeq	39.647 ± 16.677	32.808 ± 6.187	0.2306
Sleep time IOP SD before 3 a.m., mVeq	39.729 ± 20.588	25.244 ± 8.132	0.1250
Awake time IOP SD, mVeq	103.148 ± 60.864	94.902 ± 44.280	0.3716
Awake time IOP SD before 3 p.m., mVeq	36.601 ± 6.086	31.726 ± 14.034	0.1305
Difference between sleep time average IOP and awake time average IOP, mVeq	118.296 ± 46.641	126.745 ± 54.248	0.6553
Amplitude of cosine fit curve, mVeq	122.184 ± 67.493	116.234 ± 60.533	0.4136
Sleep time AUC of cosine fit curve/ID interval number	297.668 ± 112.092	205.650 ± 124.905	0.0570
GAT, mmHg	13.167 ± 2.714	12.333 ± 1.751	0.1446

* Statistically significant. Data are presented as mean ± SD. Abbreviations: AUC, area under the curve; CLS, contact lens sensor; GAT, Goldmann applanation tonometry; SD, standard deviation; IOP, intraocular pressure.

**Table 3:** The percent difference between the two CLS measurements after ripasudil eye drops administration in 2 cases.

CLS Parameter	Case Number 1	Case Number 3	Average
Awake-time maximum, mVeq	71.5/383.95 (18.6%)	134.3/335.55 (39.9%)	29.25%
Awake-time fluctuation, mVeq	194.8/445.6 (43.7%)	153.9/399.45 (38.5%)	41.1%
Sleep-time maximum, mVeq	24.6/352.5 (7%)	56.1/311.35 (18.0%)	12.5%
Sleep-time fluctuation, mVeq	37.1/125.35 (29.6%)	21/99.1 (21.2%)	25.4%

(The different values between the two measurements/the mean value of the two measurements) × 100%. Average percentage = (Case 1 percent difference + Case 3 percent difference)/2.

**Table 4:** The comparison between the CLS measurements and i care ic200 measurements after ripasudil eye drop administration in 2 cases.

	Case Number 1	Case Number 4
Pearson’s correlation between CLS IOP and ic200 IOP	0.370	0.558
Circadian IOP fluctuation of ic200, mmHg	5.9	4.1
Amplitude of cosine fit curve of CLS, mVeq	184.13	109.52
Circadian IOP fluctuation of ic200/amplitude of CLS	0.032	0.037

## Data Availability

The data are saved in archives which are managed and protected by the Department of Ophthalmology and Visual Science, Yokohama City University Graduate School of Medicine. In principle, the data are not publicly available.
